# Supplementary material for: “It’s because they’re my kids, and I love them”: Substance use disorders’ impact on children and families: A secondary analysis
Source: PLoS One. 2025 Jul 16;20(7):e0327654. doi: 10.1371/journal.pone.0327654 (PMC12266456; doi:10.1371/journal.pone.0327654)
Supplement: Attachment — Submitted filename: Response to Reviewers June 2025.docx [file pone.0327654.s004.docx]

**Response to Reviewers**

| **Reviewer Feedback** | **Author Response** |
| --- | --- |
| 1. If the authors have adequately addressed your comments raised in a previous round of review and you feel that this manuscript is now acceptable for publication, you may indicate that here to bypass the “Comments to the Author” section, enter your conflict of interest statement in the “Confidential to Editor” section, and submit your "Accept" recommendation.  Reviewer #1: All comments have been addressed  Reviewer #2: All comments have been addressed  Reviewer #3: All comments have been addressed | Thank you. |
| Is the manuscript technically sound, and do the data support the conclusions?  The manuscript must describe a technically sound piece of scientific research with data that supports the conclusions. Experiments must have been conducted rigorously, with appropriate controls, replication, and sample sizes. The conclusions must be drawn appropriately based on the data presented.  Reviewer #1: Yes  Reviewer #2: Yes  Reviewer #3: Yes | Thank you. |
| Has the statistical analysis been performed appropriately and rigorously?  Reviewer #1: Yes  Reviewer #2: Yes  Reviewer #3: N/A | Thank you. |
| Have the authors made all data underlying the findings in their manuscript fully available?  The PLOS Data policy requires authors to make all data underlying the findings described in their manuscript fully available without restriction, with rare exception (please refer to the Data Availability Statement in the manuscript PDF file). The data should be provided as part of the manuscript or its supporting information, or deposited to a public repository. For example, in addition to summary statistics, the data points behind means, medians and variance measures should be available. If there are restrictions on publicly sharing data—e.g. participant privacy or use of data from a third party—those must be specified.  Reviewer #1: Yes  Reviewer #2: Yes  Reviewer #3: Yes | Thank you. |
| Is the manuscript presented in an intelligible fashion and written in standard English?  PLOS ONE does not copyedit accepted manuscripts, so the language in submitted articles must be clear, correct, and unambiguous. Any typographical or grammatical errors should be corrected at revision, so please note any specific errors here.  Reviewer #1: Yes  Reviewer #2: Yes  Reviewer #3: Yes | Thank you. |
| Review Comments to the Author  Please use the space provided to explain your answers to the questions above. You may also include additional comments for the author, including concerns about dual publication, research ethics, or publication ethics. (Please upload your review as an attachment if it exceeds 20,000 characters)  Reviewer #1: The authors are commended for satisfactorily responding to the raised comments. This is an important study that adds to the body of knowledge regarding this important issue. | Thank you. |
| Reviewer #2: My earlier concerns have been attended to, especially on ethics which are key in any human subject research studies | Thank you. |
| Reviewer #3: I understand that this paper uses data from the main study “Engaging Families to Build Healthy Communities” that is yet to be published. This is a secondary analysis aimed at exploring new questions or applying different analytical methods to gain additional insights (specifically, the ways in which substance use affected families). To avoid confusion, I suggest that the authors replace terms like "primary study" in lines 90 and 102... with "main study," as mentioned in line 98. | We have changed the term ‘primary study’ to ‘main study’ throughout the manuscript |
| The paper meets the trustworthiness criteria (credibility, transferability, dependability, and confirmability); however, the authors need to mention how saturation of data was achieved, duration of the interviews or group discussions, whether field notes were taken during interviews or group discussions. Few sentences can be added to meet the 32-item checklist COREQ (Consolidated criteria for reporting qualitative studies). | Thank you for this helpful feedback.  We note in line 144/145 that ‘All interviews were audio-recorded and transcribed verbatim, and lasted between one and two hours in length.’  We have added a note indicating that field notes were not consistently captured.  We also added the methods for determining data saturation on line 163/164. |

Comments to the Author

________________________________________

2.

________________________________________

3.

________________________________________

4.

________________________________________

5.

________________________________________

6. Review Comments to the Author

Please use the space provided to explain your answers to the questions above. You may also include additional comments for the author, including concerns about dual publication, research ethics, or publication ethics. (Please upload your review as an attachment if it exceeds 20,000 characters)

Reviewer #1: The authors are commended for satisfactorily responding to the raised comments. This is an important study that adds to the body of knowledge regarding this important issue.

Reviewer #2: My earlier concerns have been attended to, especially on ethics which are key in any human subject research studies

Reviewer #3: I understand that this paper uses data from the main study “Engaging Families to Build Healthy Communities” that is yet to be published. This is a secondary analysis aimed at exploring new questions or applying different analytical methods to gain additional insights (specifically, the ways in which substance use affected families). To avoid confusion, I suggest that the authors replace terms like "primary study" in lines 90 and 102... with "main study," as mentioned in line 98.

The paper meets the trustworthiness criteria (credibility, transferability, dependability, and confirmability); however, the authors need to mention how saturation of data was achieved, duration of the interviews or group discussions, whether field notes were taken during interviews or group discussions. Few sentences can be added to meet the 32-item checklist COREQ (Consolidated criteria for reporting qualitative studies).

________________________________________
